# A smartphone-based online platform for clinical skills training and assessment with standardized patients: platform development and pilot study outcomes

**DOI:** 10.1080/10872981.2023.2187954

**Published:** 2023-03-12

**Authors:** Surong Jiang, Huanhuan Chen, Xiaozhi Wang, Liling Chen, Binlin Luo, Lars Konge, Junjie Du, Hua Huang

**Affiliations:** a Department of Geriatric Cardiology, The First Affiliated Hospital with Nanjing Medical University, Nanjing, China; b Department of Endocrinology, The First Affiliated Hospital with Nanjing Medical University, Nanjing, China; c Department of Cardiology, The First Affiliated Hospital with Nanjing Medical University, Nanjing, China; d The First School of Clinical Medicine, Nanjing Medical University, Nanjing, China; e Department of Plastic and Burn Surgery, The First Affiliated Hospital with Nanjing Medical University, Nanjing, China; f Copenhagen Academy for Medical Education and Simulation (CAMES), Copenhagen, Denmark; g Department of Cardiovascular Surgery, The First Affiliated Hospital with Nanjing Medical University, Nanjing, China; h Medical Simulation Center, Nanjing Medical University, Nanjing, China

**Keywords:** Clinical skills training, patient-physician interview skills, online learning platform, standardized patients, medical students

## Abstract

There are limitations and difficulties in the management of traditional in-person standardized patient (SP) practice. The latest developments in online communication tools and the COVID-19 pandemic have promoted the needs for online clinical skills training objectively. However, existing commercial online platforms may not meet the requests for SP-based medical simulation. This paper described the methodology applied to develop a smartphone-based online platform for the management of clinical skills training and assessment with remote SPs, and aimed to determine whether this new platform is acceptable or useful through a pilot run in September 2020. The post-run survey including questionnaire inspired by technological acceptance model and determinants of the perceived ease of use was used to assess the acceptability and usefulness of the platform. Twenty four-year students of clinical medicine participated in the pilot study with twenty SPs and ten faculties. Data from the post-run survey showed that there was a general recognition that the platform is easy to use among all the users. Two questions regarding the usefulness of the platform showed significant differences between the SPs/faculties and the students. More SPs found the platform useful as a training method than the students did. The faculties showed more attempts than the students to use this platform for clinical skills training in the future. This smartphone-based online platform was widely accepted among the tested students, SPs and faculties, which meets the requests and challenges of the new era. It provides an effective approach for clinical skills training and assessment with remote SPs.

## Introduction

Standardized patients (SPs) were introduced more than fifty years ago to teach and assess real-world clinical problems, in a safe and well-controlled environment [1]. Since then, SPs have been globally used in teaching and evaluating the fundamental clinical practice and professionalism of medical students, such as anamnesis acquisition, physical examinations, and doctor – patient communication [2–5]. However, there are limitations and difficulties in the management of traditional in-person SP practice. SP resources are often distributed unevenly; therefore, their activities are usually limited to certain times and places. The dialogue of doctor – patient encounters can be varied due to the different levels of students and sometimes unpredictable, which makes it difficult for SPs, especially those inexperienced ones, to recall and process some essential information in feedback sessions.

The clinical education environment has never been more diverse, taking place in an increasing number of settings instead of just high-performing academic healthcare centers [6]. Online medical simulation education, particularly in SP-based learning, is becoming even more popular with the latest developments in online communication tools. For instance, online platforms of virtual SPs have been developed and cases adapted for teaching specific topics of medicine to students at various locations [7]. More recently, in response to the global threat of COVID-19, medical schools worldwide have been incorporating SP-based clinical training and assessment into online education with various approaches [8–11]. Reliable and mature platforms and applications have been recommended for teaching clinical practice online, with dedicated design or necessary adaptation [12–14]. In the interim, tele-Objective Structured Clinical Examinations (tele-OSCEs) via video conferencing, between students and SPs, have been promptly implemented, as in-person activities have been greatly reduced or completely cancelled [15,16]. However, engaging an existing commercial online platform, such as Zoom, for SP-based teaching and training, may pose security and privacy concerns, jeopardizing SP work safety norms or resulting in a leak of confidential information. For instance, thousands of Zoom conference recordings have lately been discovered online, which may include case information of medical exams [17]. Attendees’ online identifications (IDs) are exposed during a Zoom meeting, and people in the meeting room could contact each other without supervision [18].

Therefore, a more exclusive platform with a higher level of security is favored, in which personal IDs can be encrypted and attendees’ interactions are strictly regulated to the learning objectives. The main purpose of this study was to develop an online platform to avoid the abovementioned problems, by providing a solution for clinical skills training and assessment using SPs remotely. Additionally, we presented user acceptance survey data from a pilot run of this newly developed platform.

## Methods

A smartphone-based online platform, for the management of clinical skills training and assessment with SPs, was designed for medical students by faculty academicians and software engineers. First, a case library was established, and SP cases were arranged by adapting a case development template from the Association of Standardized Patient Educators (ASPE) [19]. Second, we constructed the framework of the platform, which consists of two ports and six systematic modules. Third, to facilitate the implementation of the platform, we executed a well-designed training plan for SPs, faculties, and students. Finally, a pilot run of the platform, with a post-run survey, was conducted to assess the perceived ease of use (PEOU) and perceived usefulness (PU) thereof. We present the details of the above-mentioned four stages in the following sections.

### Stage 1: case development

**Figure 1.**
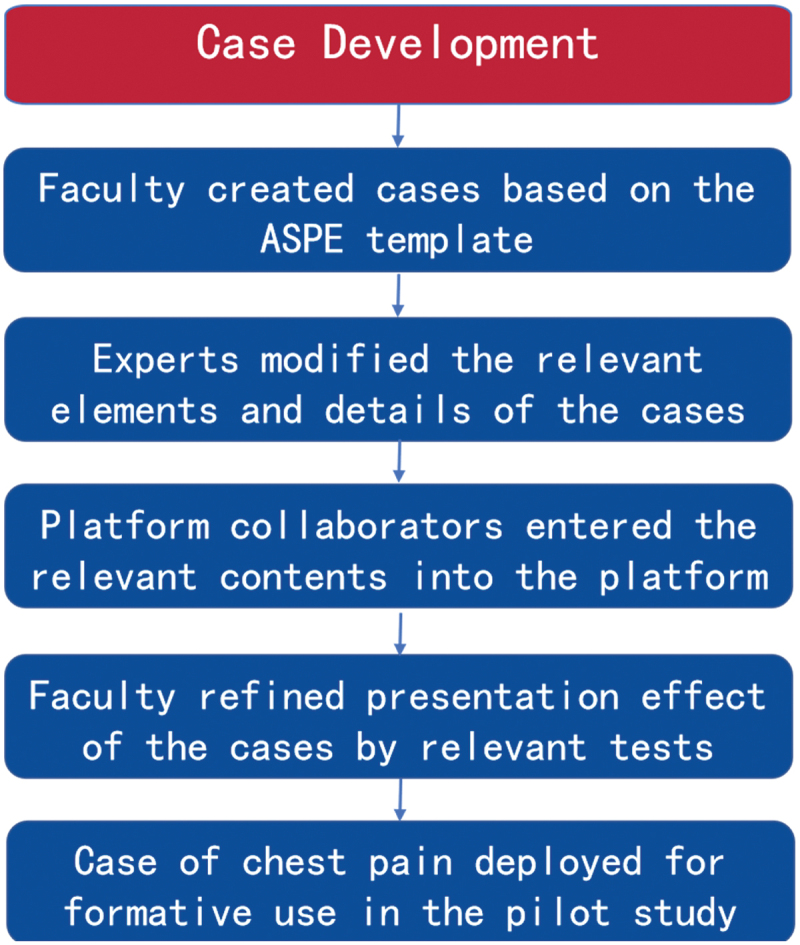
Flow diagram displaying steps taken to develop cases for the online platform.

As shown in Figure 1, our case development went through the following processes. First, cases were initially developed by the SP case development group of Nanjing Medical University, which consisted of SP educators and medical experts from a wide range of specialties. Case components were based on foundational and typical clinical symptoms. Case creators received at least basic coaching from the platform creators which included technical elements such as platform frame, screen location, audio adjustments, and computer system optimization. Case creators then wrote eight typical cases using the ASPE case development template [20]. These cases were categorized into different specialties of internal medicine and surgery, which aimed to improve the students’ skills on history-taking and clinical thinking. Second, the platform creators transferred the case contents into the platform which had been tested iteratively. Primary testing of the case was provided by at least one case creator playing the case. The platform creators and case creators then reached agreement on case contents, the overall case complexity, and evaluation method, including the number and types of evaluation items. Sometimes, case creators in relevant fields were invited to adapt the elements and details of the case to fit characteristics of the online form, such as paying more attention to the description of the patient’s facial expressions, rather than physical movement of the body. For information that could not be obtained via the online mode, a consensus was reached between case creators and platform creators that the relevant contents, such as positive signs during physical examinations, should be included in the platform and displayed at runtime via on-screen controls. Finally, prior to the pilot run of the case, relevant tests were conducted to ensure the best presentation effect of the cases on the mobile phone screen. For example, when we advocate for eye contact during online communication, we recognize that learners may be unsure about where to look on the screen. To address this issue, we worked with the platform creators to test the best placement for the patient’s face. Our testing process involved several steps. First, the SP clicked on the ‘presentation’ button on the platform, which displayed a box similar to face recognition. Then, the SP adjusted the camera to fit their face into the frame as much as possible. If the platform did not recognize the face, a text prompt was given. If the identification was successful, the process proceeded to the next step. After testing, we found that placing the patient’s face in the middle of the screen and minimizing distractions, such as their own image or other information in the bottom corner of the screen, was the most effective way to promote eye contact and engagement during online communication. Of note, some technical issues with the tele-communications tools have also been improved during tests, such as poor connection or audio/visual delays. Using the process described above, we have created four cases for use in the platform: jaundice, dyspnea, abdominal pain, and chest pain. The case scenario instructed the learners:

- to gather a thorough medical history by communicating with the SP via the online platform;
- to compare and distinguish features of a SP’s clinical problem presentation to delineate likely causes of the symptoms during online doctor – patient encounters;
- to develop an appropriate, prioritized differential diagnosis with supportive evidence from the medical history and provide physical examination findings; and
- to formulate medical plans for treatment, including both immediate and follow-up plans.

Thus, with the developed online SP cases, we can train the students in anamnesis acquisition and clinical reasoning, including the identification of patients’ symptoms, patient data organization, differential diagnosis prioritizing, and choice of therapeutic principles. Of note is doctor – patient communication skills are also focused upon as learning objectives.

### Stage 2: Framework Construction

We constructed a systematic framework to enable the online training and assessment for medical students in Nanjing Medical University. This was done by software engineers in cooperation with SP educators and faculties.

**Figure 2.**
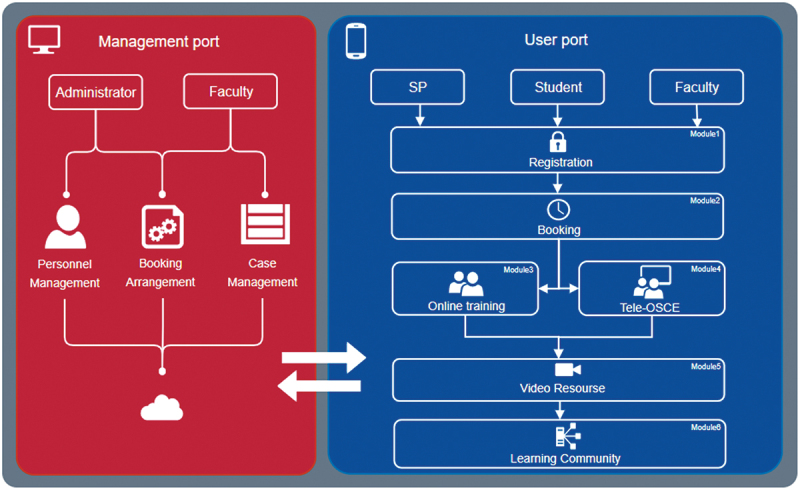
A schematic showing the platform framework.

The online platform can be divided into two ports. The management port is built on a website and is operated by administrators and faculties, for the management of personnel, cases, and booking information of training or assessment. The user port is a WeChat Mini Program, which is a JavaScript Application Programming Interface that users can efficiently access using smartphones, without downloading or installing any other applications, providing that they all have WeChat installed on their smartphones. The user port has different user interfaces (UIs) for students, SPs, and faculties as different UIs fit for separate roles and functions. As shown in the Figure 2, in the user port, there are six functional modules: User registration/login, Booking, Online training, tele-OSCE, Video resource, and Learning community.

To use the platform, registration and ID verification are required in advance. Once permitted by the administrators, users can log in to the platform with their registered phone number at any time. The administrators and faculties upload case information, such as topics (chief complaint) and learning schedules, into the Booking module, according to the teaching calendar or training plans. Then SPs decide and publish their personal available times within that period of schedule, which enables the students to choose the suitable time slot, whenever they are ready for the training. When the booking process is completed, an appointment confirmation text will be sent automatically to the student and the chosen SP.

In the Online training module, the SP will have a live video consultation with the students in a virtual room, according to the arrangement in the booking module. In the interim, faculties can observe the training by logging into the platform and entering the same virtual room. Faculties can converse with the student or the SP during the process, to give instructions or feedback. The whole training process will be recorded and saved automatically. Students and SPs can review the videos in the video resource module. Faculties can evaluate the performance of SPs and students by leaving comments in the comment section of the video resource module and decide whether to let the SPs and students see these comments or not.

The tele-OSCE module works slightly differently from the online training module. Usually, faculties arrange an examination time for a tele-OSCE which will be agreeable for all participants. At the examination time, the student will be waiting at a virtual waiting room, to be sent to the station of the tele-OSCE, when the SP and examiners are ready to begin. The examiner judges the student’s performance in real time, using an electronic checklist on the screen provided by the tele-OSCE module, while the entire process is recorded synchronously, to realize the traceability of the test process. A score will be generated and uploaded automatically to the management port of the platform once the examination is finished, as well as when the checklist is completed.

The last module is called Learning community. In this module, it provides users an active learning environment where more learning materials, such as videos of relevant lectures and useful weblinks, are available. Furthermore, students can communicate with each other in this module and share their learning experience or send questions to SPs and faculties, who may leave answers or comments in the discussion section of the module.

### Stage 3: personnel training

#### SP Training

All SPs were recruited by Nanjing Medical University. They were trained by our SP educators who have been trained and qualified by the ASPE. The learning materials for SPs are based on the cases from the case library. SPs are guided to the specific contents, skills, concerns, and physical behaviors of the patient in the case. It is also important to include software engineers on the SP training course, to help them become familiar with the platform, including how to register and log in, publish available training times, start a conversation with students via the platform, play back the videos, leave comments, and so on.

#### Faculty training

Teachers and examiners are experts with different clinical specialties who are familiar with the symptoms of the cases. They would be trained on the case materials, learning or assessment objectives, and platform-related operations. They also learn how to deal with the technical problems of the platform, how to answer questions from students, and leave comments on the platform. This is to ensure that the online learning/assessment proceeds smoothly in a standardized manner with minimal technical difficulties.

#### Student training

Students receive training prior to using the platform. Their training includes reading a written introduction and viewing a demonstration video on how to use the platform, prior to viewing a simulated simple demo case. They also learn how to manage emergency situations with standard protocol when using the platform.

### Stage 4: pilot run

#### Participants

The pilot run learners were fourth-year students in clinical medicine who were supposed to go to hospitals to conduct their clerkship practice but had to remain in school due to the COVID-19 pandemic in 2020. The inclusion criteria were: 1) students have completed the curricula of Diagnostics and Internal Medicine; 2) they have a smartphone that can be used for the study; 3) they have not taken part in any form of online training of clinical skills; and 4) they are willing to participate in the pilot run. Prior to joining in, these student participants were informed that the pilot run results would not have any impacts on their course score records and they could withdraw at any time. The study design was approved by the ethics committee of Nanjing Medical University and the informed consent was not required.

#### Case information for the pilot run

For the pilot run, we decided to use a case of a patient presenting with chest pain, as it is one of the most important cardiovascular complaints commonly seen in the primary care setting. The complete list of case information is available in the student worksheet (Appendix A), SP case training material (Appendix B), and student checklist and score sheet (Appendix C). The learning objectives of this training activity were to allow students to practice anamnesis acquisition with SPs and give initial diagnosis of the case and make at least three differential diagnoses.

#### Procedures of the pilot run

One week prior to the pilot run, we emailed students a brief overview of the assignment. Meanwhile, SPs were asked to publish their own available times for the training session, and students then chose a time slot to make an appointment accordingly.

The training process lasted for approximately 20 minutes. The students spent the first 10 minutes talking to the SPs to obtain information regarding the patient’s medical history. Next, the student was asked to type the answers to the questions, including providing preliminary diagnosis and giving three differential diagnoses, and upload to the platform. After that, the SP would start an oral feedback process with the student, using the feedback sandwich technique [21]. The feedback would focus on the student’s communication skills and professionalism.

When the training was completed, the SPs were asked to complete and upload an SP’s checklist to the platform for the student to read. Faculties would choose to observe the students either by entering the virtual room as the same time as the training process, or by watching the replay video from the video resource module at a later stage. Either way, faculties were asked to complete a teacher’s checklist which focuses on the content integrity and logic of the consultation and upload it to the platform for the students to read. Students were encouraged to replay the video and check the feedback on the video resource module and leave messages to communicate with SPs and faculties on the learning community module if they have further questions.

### Post-run survey on user acceptance of the platform

We used an online anonymous survey to evaluate the acceptability of this new online platform, through the perspectives of students, SPs, and faculties after they finished the test training process. To assess user acceptance of the platform, the survey used Technology Acceptance Model and determinants of the PEOU [22,23]. All participants received a short message on their mobile phones at the end of the pilot run, which contains a link to a questionnaire. The questionnaire included 10 questions based on a 5-point Likert scale. Among these questions, five of them assess whether the online platform is easy for participants to use, namely PEOU. Another five questions assess PU and intention to use the platform, which reveled whether the platform has an impact on their performance and whether they are willing to continue to use it in the future.

### Statistical analysis

Descriptive statistics were used to analyze the user acceptance of the platform by students, SPs, and faculties. The mean rating scale of each item of the survey done by the three groups were calculated and compared using one way ANOVA with Tukey’s multiple comparisons test for the chosen two groups. A *p*-value less than 0.05 is considered statistically significant.

## Results

We enrolled 20 students, who were randomly selected from eligible candidates, in September 2020, to participate the pilot study, including eight men and 12 women. The pilot study used 20 female SPs, as they had the same sex as the patient in the case. We asked 10 faculties to join the study who had at least five years of teaching experience on anamnesis acquisition and clinical thinking. Neither the SPs nor the faculties had experience in using this kind of online training platform. All participants in the study completed the questionnaires shortly after they finished the test training process. The questionnaire revealed a Cronbach’s alpha of 0.973 (*n* = 10 items), and item-total correlation ranged between 0.785 and 0.928.

**Figure 3.**
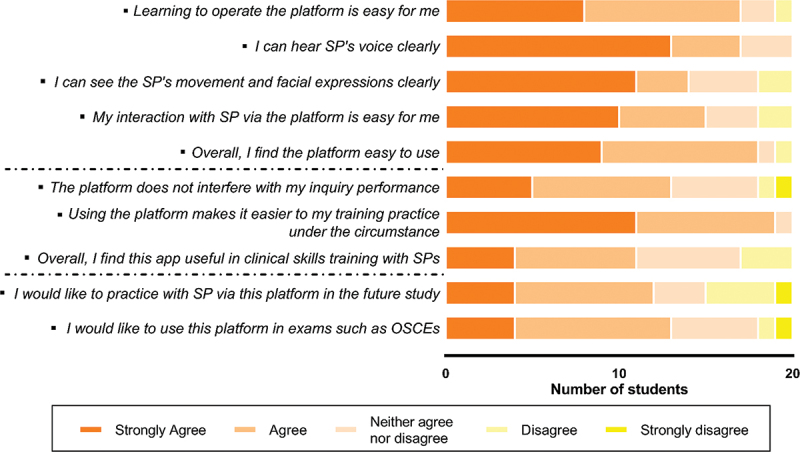
Acceptability of the platform by the students.

According to the students’ responses of the survey, 90% (*n* = 18) agreed or strongly agreed that the platform was generally easy to use, and 55% (*n* = 11) found it useful in clinical skills training with SPs. In Figure 3, 65% (*n* = 13) said they would like to use the platform for future studies or exams that are associated with doctor-patient encounters.

**Figure 4.**
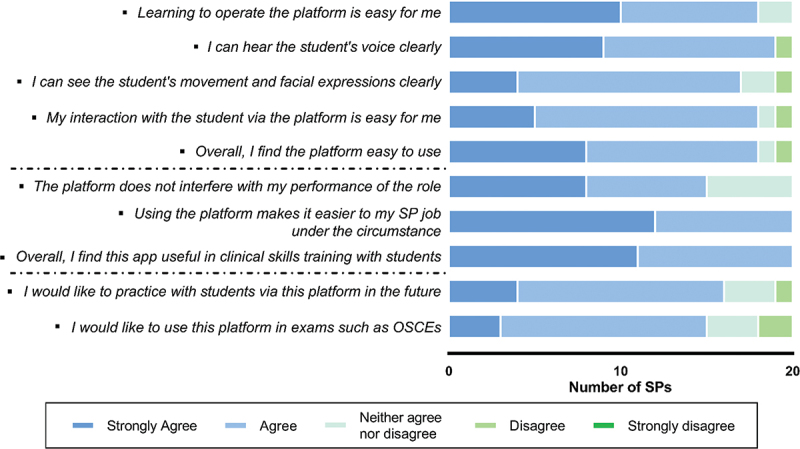
Acceptability of the platform by the standardized patients.

We also invited all 20 SPs to write down their opinions after the trial run. It was reported that 90% (*n* = 18) noted the online platform was easy to use, as only 5% (*n* = 1) SP disagreed on whether they could hear and see the students clearly. All (*n* = 20) agreed or strongly agreed that this was useful for clinical training with students. Majority of SPs (80%, *n* = 16) would like to use the online platform for teaching and 75% (*n* = 15) of them were willing to implement the online platform to assess students in the future (Figure 4).

**Figure 5.**
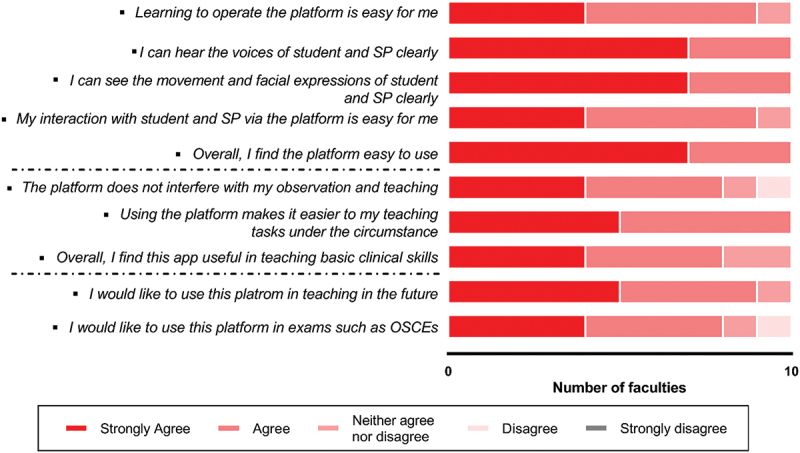
Acceptability of the platform by the faculties.

Six faculties observed the live training, while another four watched the replay videos. All faculties (*n* = 10) reported that they could hear and see the conversations clearly and thought the software was easy to use. There was only one faculty (10%) who considered that observing students via devices could affect their judgement. The majority of the faculties (90%, *n* = 9) accepted the online platform for teaching, and 80% (*n* = 8) would likely recommend this form for the purpose of assessment in the future (Figure 5).

Finally, a one-way ANOVA was performed to compare the average rating scales of the three groups on each item of the survey (Table 1). In general, the faculties and the SPs held more positive opinions than the students.

**Table 1.** Average rating scores of survey questions by all participants.

Survey questions	Average rating scores (M ± SD)		
	Students (20)	SPs (20)	Faculties (10)
Learning to operate the platform is easy for me.	4.20 ± 0.83	4.40 ± 0.68	4.30 ± 0.67
I can hear SP’s/student’s voice clearly.	4.50 ± 0.76	4.35 ± 0.75	4.70 ± 0.48
I can see the SP’s/student’s movement and facial expressions clearly.	4.15 ± 1.09	4.00 ± 0.73	4.70 ± 0.48
My interaction with SP/student via the platform is easy for me.	4.15 ± 1.04	4.10 ± 0.72	4.30 ± 0.67
Overall, I find the platform easy to use.	4.30 ± 0.80	4.25 ± 0.79	4.70 ± 0.48
The platform does not interfere with my inquiry performance/role play/observation.	3.75 ± 1.07	4.15 ± 0.81	4.10 ± 0.99
Using the platform makes it easier to my training/teaching practice under the circumstance.	4.50 ± 0.61	4.60 ± 0.50	4.50 ± 0.53
Overall, I find this app useful in clinical skills training/teaching with SPs.	3.60 ± 0.99	4.55 ± 0.51#	4.20 ± 0.79
I would like to practice with SP/students/teaching via this platform in the future study.	3.50 ± 1.19	3.95 ± 0.76	4.40 ± 0.70*
I would like to use this platform in exams such as OSCEs.	3.70 ± 1.03	3.80 ± 0.83	4.10 ± 0.99

NOTE: #*p* < 0.01 compared with the students’ group. *p < 0.05 compared with the students’ group. *M* = mean, SD = standard deviation.

There was a statistically difference in mean rating scales of the 8^th^ item between at least two groups (F (2, 47) = 7.369, *p* = 0.0016). Tukey’s HSD Test for multiple comparisons found that, compared with the students, more SPs found the platform useful as a training method (*p* = 0.001, 95% C.I. = [−1.553, −0.347]). There was no statistically significance in mean rating scales between students and faculties (*p* = 0.132) or between SPs and faculties (*p* = 0.491).

There was a statistically difference in mean rating scales of the 9^th^ item between at least two groups (F (2, 47) = 3.151, *p* = 0.036). Tukey’s HSD Test for multiple comparisons found that the faculties showed more efforts to use this platform for clinical skills training in the future than the students (*p* = 0.047, 95% C.I. = [−1.789, −0.011]). There was no statistically significance in mean rating scales between students and SPs (*p* = 0.300) or between SPs and faculties (*p* = 0.445).

## Discussion

It has become a hot topic in healthcare simulation education to use the latest network technology to integrate reliable resources and improve learning and assessment methods [24–26]. Since the outbreak of COVID-19, an increasing number of academic institutions have turned to teaching clinical skills online, as this is the most feasible approach, given the circumstances. Accordingly, we developed a smartphone-based online platform to facilitate interactions among medical students, SPs, and medical teachers via the Internet. This virtual platform provides a smooth and secure experience of online training of anamnesis acquisition and doctor – patient communication and serves as an alternative approach to the issue of in-person clinical skills training during special times, such as the COVID-19 pandemic. With the development of the cases and core modules, the platform can be used for summative assessment when in-person OSCE is not possible.

The platform offered the following advantages. First, the platform blends learning, assessment, and management into a comprehensive, reliable, visual, and interactive interface for training and assessment. Second, this platform, which is built on smartphone applications, can realize the goal of ubiquitous learning. Students have access to information and can communicate with one another at any time and from any location. Third, SPs decide and publish training times, and students select training cases and times to be trained. In Addition, students can obtain feedback from SPs and tutors through the platform, which may lead to positive behaviors of students, such as better engagement, initiative, and potential learning effectiveness [27]. Last but not least, this WeChat-Mini-Program-based smartphone application provides a securer platform than those existing commercial ones. Users’ IDs are encrypted with unified codes, e.g., student 1 and patient 2. Only authorized administrators and faculties can access their personal information from the management port. Students or SPs cannot contact each other without supervision via the platform. The video resources can be reviewed online within the platform but they are not allowed to download technically.

Data from the pilot run showed surprisingly high PEOU from the students, SPs, and faculty members, as there was a general recognition among all the users that the platform is easy to use. Most students could observe the subtle facial expressions and body movements of SPs, as they could see and hear each other clearly during the conversations. Only one or two students did not have valuable experience during online conversations, which turned out to be attributed to poor network connectivity. Indeed, one of the most common challenges of online learning lies with inadequate capacity of Internet servers or IT services causing network congestions or crashes, rather than other barriers [24,28,29]. Although the development of a new generation of networks and more powerful electronic devices can minimize these problems, foreseeing the potential issues of network connection, preparing for the worst, and most importantly, testing the testable aspects and troubleshooting the systems in advance, may improve the user experience [30].

Different user groups had various opinions towards the PU of the online platform. Although all users, except one student, strongly agreed or agreed that using the platform made it easier for their learning/teaching tasks than the traditional way during the COVID-19 pandemic, just over half of students considered it useful for clinical skills training. Interestingly, these medical students are among the first generation of “digital natives” who grew up surrounded by digital technology and therefore feel more comfortable processing digital information than the SPs and faculties do [31]. This relatively less positive opinion among students could be because this new form has not formally been accounted for in their assessment as students and which are largely result-orientated [24]. Conversely, most SPs considered that the platform had no influence on their performance during the online conversations and all of them recommended it as useful for clinical skills training. This overwhelming welcome by SPs may be thanks to the user interface design of the platform, which followed the principle of the Standards of Best Practice recommended by the Association of Standardized Patient Educators [20]. In this way, the online activities of SPs, such as the role portrayal, should not be physically or cognitively challenged, in this case, by the operation of the platform or by the different feelings in a virtual space [32]. Nevertheless, based on their general positive experience with the platform, most students and SPs were confident to engage with this new form of training and enthusiastic about using this approach in the future.

One limitation of this study lies in that only a small group of students, SPs and faculties participated in the post-run survey, which makes the POEU and PU results less convincing. The next stage is to implement this online training in the regular curricula of third-year medical students at our university, to confirm its teaching effectiveness on a larger scale. The utilization of the platform on formative assessment, such as tele-OSCE, also requires evaluation. In fact, our team members have administered a tele-OSCE to 176 candidates in a high-stakes test in March 2021 using this platform with satisfactory results.

## Conclusion

This smartphone-based online platform was widely accepted among the tested students, SPs, and faculties, which met the requests and challenges of the new era. It provides an effective approach for clinical skills training and assessment with remote SPs. The compilation of cases and personnel preparations are crucial to the smooth participation of students and SPs in the training and assessment process on the platform. Future research should prove its applicability to a broader set of users.

## Data Availability

The data that support the findings of this study are available on request from the corresponding author, JD. The data are not publicly available due to restrictions e.g., their containing information that could compromise the privacy of research participants.

## Appendices

After the training begins, the screen will display the following contents in sequence:

### Tasks to be completed

**1. History taking**:

Please elicit a complete medical history for this patient.

**2. Clinical reasoning**:

You will have an on-line test based on the medical history. Please answer questions in text one by one.

### Patient encounter length

1. 10 minutes for task 1
2. 10 minutes for task 2

**Patient Name:**

Zhenping Han

**Age**: 48 years old

**Gender**: Male

**Chief Complaint**: Chest pain

**Vital Signs**: **(if applicable)**

Blood Pressure: 100/72 mmHg

Temperature: 36.5°C

Respiratory Rate: 15 breaths per minute

Heart Rate: 55 beats per minute

BMI: 24.9 Kg/m^2^

## Lab Results: (if applicable)

CBC: WBC 10.54 × 10^9^/L, N 70%, Hb 138 g/L, PLT 293 × 10^9^/L;

Biochemical test: ALT 41.6 U/L, AST 196.8 U/L, CK 434.6 U/L, CK-MB 67.8 U/L, Cr 66.7umol/L;

NT-proBNP: 676 pg/mL;

cTnT: 1360ng/L;

D-Dimer:1.05 mg/L

**Image Results: (if applicable)**

The ST segment in leads II, III, aVF, and V3-5 R is significantly elevated, and the ST segment in leads I and aVL is depressed.

## Appendix B. SP Case Training Material

### Part 1 – Administrative Details

**Patient Name:**

Zhenping Han

**Patient’s Reason for the Visit:**

Find the cause and relieve the symptoms

**Patient’s Chief complaint:**

Chest pain

**Differential Diagnosis:**

Aortic dissection; Pulmonary embolism; Spontaneous tension pneumothorax; Acute pericarditis

**Actual Diagnosis:**

Acute myocardial infarction

**Case Purpose or Goal:**

Summative learner practice

**Level of the learner and discipline:**

Fourth year clinical medicine students

**Learner’s prerequisite knowledge and skills:**

Completed course on Internal Medicine and Diagnostics

**Case authors:**

Xiaozhi Wang, Miao Lu,Jian Zhang, Juanjuan Tang, Wei Sun, Huaxin Huang, Surong Jiang

**Date of case development: **

01/10/2021

**Summary of patient story:**

Mr. Zhenping Han, 48 years old, had recurrent chest pain half a day ago. The left chest pain is accompanied by left shoulder and back pain during the attack, which lasted 5–10 minutes each time and was not significantly related to activities. There were 1–2 episodes a day, mostly at night, but occasionally during the day. Half a day ago, his chest pain persisted and could not be relieved. For further treatment, he went to the outpatient clinic of the hospital. After oral medication, the symptoms did not improve significantly. For further treatment, he was admitted to the hospital.

**Learning/Case objectives**:

1. Employ hypothesis-driven history taking to identify key or distinguishing features of a patient’s clinical presentation during standardized patient encounters.
2. Interpret related inspection results, such as electrocardiogram finding, to further characterize a patient’s problem representation.
3. Formulate an appropriate, prioritized differential diagnosis by comparing and contrasting the patient’s problem representation with one’s illness scripts for cardiovascular conditions.
4. Demonstrate Cohen/Cole communication skills (at least 5 items).

**List of assessment instruments used: (e.g., SP checklist, post-encounter notes, quiz)**

SP Checklist.

Post-encounter quiz.

**Event format: (e.g., formative, summative, small group, individual, multi-station OSCE, duration)**

Summative.

OSCE.

**Demographics of patient/recruitment guidelines:**

Age: 35–60 years old.

Gender: Male.

Ethnicity: Chinese.

Body size: Normal or slightly fat.

**List of special supplies needed: (e.g., additional materials *see part 6*, moulage, props, SP attire, etc.)**

Sphygmomanometer, stethoscope, SP wears casual clothes.

### Part 2 – Contents for SPs

**Presentation and Resulting Behaviors**

**Affect**: nervous and anxious, not cooperative in answering questions.

**Body language**: press the precordial area with the right hand, bent over when sitting down.

**Facial expression**: painful.

**Eye contact**: tired of communication.

**Opening Statement**

I have been having chest pain.

**History of Present Illness (HPI): (consider the following)**

**Quality/Character**

Pressure-like, colicky, squeezing sensation.

**Onset**

The first episode of chest pain was about 3 months ago, and I have been having it from half a day ago.

**Duration**

During the 3 months, it was paroxysmal. Half a day ago it became persistent.

**Location**

Precordial.

**Radiation**

It also seems to travel to my left shoulder and back.

**Intensity**

On a scale of 1-10, it feels like a 6. Half a day ago it became 8/10.

**Aggravating Factors**

It came on with exertion or stress.

**Alleviating Factors**

It went away if I had a rest for about 5 minutes. Half a day ago it had no relief.

**Associated Symptoms**

Chest tightness.

**Past Medical History (PMH): (consider the following)**

**Illnesses/Injuries**

Hypertension.

**Hospitalizations**

None.

**Surgical History**

None.

**Screening/Preventive**

N/A.

**Medications (Prescription, Over the Counter, Supplements)**

No history of antihypertension consumption.

**Allergies (e.g., environmental, food, medication, and reaction)**

None.

**Gynecologic History (if relevant)**

None.

**Family Medical History: (consider the following)**

**Relevant Conditions/Chronic Diseases (management/treatment)**

No history of chronic cardiac disease in his family.

**Social History:**

**Substance Use (past and present)**

**Drug Use (Recreational and medications prescribed to other people)**

None.

**Tobacco Use**

20 cigarettes/day for 20 years.

**Alcohol Use**

None.

**Home Environment**

Lives in a single-family home.

**Occupation**

Staff, often work overtime.

**Leisure Activities**

You enjoy spending time with your friends and family travelling.

**Diet**

You eat a lot of easy to prepare foods.

**Exercise**

You do not exercise routinely.

**Physical Exam Findings: (may also include instructions on replicating findings)**

Vitals:

Temperature: 36.5℃; Blood pressure: 100/72mmHg; Heart rate: 55 beats per minute; Respiratory rate: 15 breaths per minute

Sinus bradycardia, regular, no murmur or rubs.

## Appendix C. Student Checklist and Score Sheet

### Part 1. History Taking

Name: Date: SP:

**Table:**

History taking		
Basic information:		
1. Patient’s name (‘*Zhenping Ha*n’).	Yes	No
2. Patient’s gender (‘*male*’).	Yes	No
3. Patient’s age (‘*48*’).	Yes	No
Questions for chest pain:		
4. Patient’s reason for the visit (‘*chest* pain’).	Yes	No
5. Onset of chest pain (‘*three months ag*o’).	Yes	No
6. Timing and frequency of chest pain episodes (‘*1–2 episodes a day, mostly at night, but occasionally during the day*’).	Yes	No
7. Causes of chest pain (‘*feel uncomfortable for no reason and no obvious correlation with activities*’).	Yes	No
8. Location of chest pain (‘*pr*ecordial’).	Yes	No
9. Scope of chest pain (‘*about the size of a palm*’).	Yes	No
10. Quality of chest pain (‘*colicky* pain’).	Yes	No
11. Duration of chest pain (‘*5–10 minutes* each time’).	Yes	No
12. Radiation of chest pain (‘*travel to left shoulder and back*’).	Yes	No
13. Alleviating factors (‘*have a rest*’).	Yes	No
14. Aggravating factors (‘*half a day ago it had no relief*’).	Yes	No
15. Associated symptom ‘chest tightness’ (‘*pressure-like*’).	Yes	No
16. Other associated symptoms such as ‘fever, chills, asthma, fatigue, nausea, vomiting, abdominal pain, diarrhea, cough, sputum, hemoptysis’ (‘*none*’).	Yes	No
17. Medical treatment and medication measures (‘*none*’).	Yes	No
General condition since onset:		
18. Mental state (*‘normal if there is no chest pain’*).	Yes	No
19. Diet (‘deteriorated appetite’).	Yes	No
20. Urination and bowel movements (‘the same as usual’).	Yes	No
21. Sleeping (‘*the same as usual*’).	Yes	No
Past medical history:		
22. Similar symptoms in the past *(‘none’*).	Yes	No
23. Chronic diseases such as ‘hypertension’, ‘diabetes’, ‘coronary heart disease’*(‘hypertension for 1 year’).*	Yes	No
24. Allergies *(‘none’).*	Yes	No
Social History: 25. Alcohol use *(‘none’).*	Yes	No
26. Tobacco use (‘20 cigarettes/day for 20 years’).	Yes	No
27. Family medical history *(‘none’).*	Yes	No

**Table:**

Grading scale (total score 50 points) No. 18~21 (Yes = 1, No = 0) (4 points). No. 1~17 and 22~27 (Yes = 2, No = 0) (46 points).

Doctor – patient communication (total score 10 points)

1. Build a relationship (The doctor expresses concern in their words, uses less medical jargon, and can increase my confidence in the treatment).
  - Extremely effective (2.0)
  - Quite effective (1.5)
  - Moderately effective (1.0)
  - Slightly effective (0.5)
  - Not effective at all (0)
2. History-Taking skills (The doctor’s inquiries are organized and can be used step by step, alternately using open-ended and closed-ended questions, and a staged summary of what I have said).
  - Extremely effective (2.0)
  - Quite effective (1.5)
  - Moderately effective (1.0)
  - Slightly effective (0.5)
  - Not effective at all (0)
3. Listen patiently (The doctor listens attentively to my language messages and rarely interrupts me).
  - Extremely effective (2.0)
  - Quite effective (1.5)
  - Moderately effective (1.0)
  - Slightly effective (0.5)
  - Not effective at all (0)
4. Be considerate of my feelings (The doctor can be considerate of my current painful feelings, corresponding to my emotions, and try to find ways to relieve my pain and anxiety).
  - Extremely effective (2.0)
  - Quite effective (1.5)
  - Moderately effective (1.0)
  - Slightly effective (0.5)
  - Not effective at all (0)
5. Satisfy the needs (The doctor was able to convey the information clearly and provide guidance on how we should proceed).
  - Extremely effective (2.0)
  - Quite effective (1.5)
  - Moderately effective (1.0)
  - Slightly effective (0.5)
  - Not effective at all (0)

### Part 2. Clinical Reasoning

Name: Date: Teacher:

**Table:**

Question 1: What is the most likely diagnosis for this patient? Please briefly list the evidence for diagnosis (total score 10 points)		
Primary diagnosis:	false	true
Mention: Coronary Atherosclerotic Heart Disease	0	1
(2) Mention: Acute ST-segment elevation myocardial infarction	0	1
(3)Mention: Inferior myocardial infarction	0	1
(4) Mention: Combined right ventricular myocardial infarction	0	1
Diagnosis points:		
(5) Mention: Symptoms of typical chest pain episodes in the past three months (point 1).	0	1
(6) Mention: Half a day ago, the symptoms were aggravated and continued to be unable to be relieved.	0	1
(7) Mention: Myocardial enzyme (Point 2) (AST 196.8 U/L ; CK 434.6 U/L; CK-MB 67.8 U/L).	0	1
(8) Mention: cTNT 1360ng/L.	0	1
(9) Mention: auxiliary examination (point 3): ECG changes.	0	1
(10) Mention: the ST segment of ECG leads II, III, aVF and V3-5 R is significantly elevated	0	1

**Table:**

Question 2: Which diseases need to be differentially diagnosed and briefly describe the main points of the differential diagnosis (total score 16 points)		
Differential diagnosis	false	true
Aortic dissection	0	2
(2) Aortic dissection: Chest pain is severe, often radiating to the back, ribs, abdomen, waist, and lower extremities. The blood pressure and pulse of the two upper extremities may be different. Two-dimensional echocardiography and CTA of the thoracic and abdominal aorta are helpful for diagnosis.	0	2
(3) Acute pulmonary embolism	0	2
(4) Acute pulmonary embolism: Patients often have chest pain, hemoptysis, dyspnea, cyanosis, and jugular vein filling. The electrocardiogram may have SIQIIITIII signs, T-wave inversion in the right chest lead and other changes. Hypoxemia is often present, and pulmonary artery CTA and radionuclide lung ventilation-perfusion scan can help identify.	0	2
(5) Spontaneous tension pneumothorax.	0	2
(6) Spontaneous tension pneumothorax: Acute onset due to coughing, weight-bearing, strenuous exercise, etc. Chest pain is on one side, just like a knife cutting. Chest X-ray can confirm the diagnosis.	0	2
(7) Acute pericarditis.	0	2
(8) Acute pericarditis: There may be severe and persistent precordial pain which may be aggravated by breathing and coughing, accompanied by fever and early pericardial friction sounds. Except for aVR, the other leads of the ECG all have ST-segment arched elevation.	0	2
(9) Other reasonable differential diagnosis 1 (including disease name and key points of differential diagnosis).	0	4
(10) Other reasonable differential diagnosis 2 (including disease name and key points of differential diagnosis).	0	4
(11) Other reasonable differential diagnosis 3 (including disease name and key points of differential diagnosis).	0	4
(12) Other reasonable differential diagnosis 4 (including disease name and key points of differential diagnosis).	0	4
